# Test-retest reliability of knee kinesthesia in healthy adults

**DOI:** 10.1186/1471-2474-8-57

**Published:** 2007-07-03

**Authors:** Eva Ageberg, Johan Flenhagen, Jonatan Ljung

**Affiliations:** 1 Division of Physiotherapy, Department of Health Sciences, Lund University, Lund Sweden; 2 Department of Physiotherapy, Blekinge Hospital, Karlskrona, Sweden; 3 Department of Physiotherapy, Samrehab Skene Hospital, Skene, Sweden

## Abstract

**Background:**

Sensory information from mechanoreceptors in the skin, muscles, tendons, and joint structures plays an important role in joint stability. A joint injury can lead to disruption of the sensory system, which can be measured by proprioceptive acuity. When evaluating proprioception, assessment tools need to be reliable. The aim of this study was to assess the test-retest reliability of a device designed to measure knee proprioception.

**Methods:**

Twenty-four uninjured individuals (14 women and 10 men) were examined with regard to test-retest reliability of knee kinesthesia, measured by the threshold to detection of passive motion (TDPM). Measurements were performed towards extension and flexion from the two starting positions, 20 degrees and 40 degrees knee joint flexion, giving four variables. The mean difference between test and retest together with the 95% confidence interval (test 2 minus test 1), the intraclass correlation coefficient (ICC_2,1_), and Bland and Altman graphs with limits of agreement, were used as statistical methods for assessing test-retest reliability.

**Results:**

The intraclass correlation coefficients ranged from 0.59 to 0.70 in all variables except one. No difference was found between test and retest in three of the four TDPM variables. TDPM would need to decrease between 10% and 38%, and increase between 17% and 24% in groups of uninjured subjects to be 95% confident of detecting a real change. The limits of agreement were rather wide in all variables. The variables associated with the 20-degree starting position tended to have higher intraclass correlation coefficients and narrower limits of agreement than those associated with 40 degrees.

**Conclusion:**

Three TDPM variables were considered reliable for observing change in groups of subjects without pathology. However, the limits of agreement revealed that small changes in an individual's performance cannot be detected. The higher intraclass correlation coefficients and the narrower limits of agreement in the variables associated with the starting position of 20 degrees knee joint flexion, indicate that these variables are more reliable than those associated with 40 degrees. We, therefore, recommend that the TDPM be measured with a 20-degree starting position.

## Background

Sensory information from mechanoreceptors in the skin, muscles, tendons, and joint structures plays an important role in joint stability [1-4]. The sensorimotor system covers the whole process from a sensory stimulus to muscle activation, i.e., acquisition of a sensory stimulus and conversion of the stimulus into a neural signal, transmission of the neural signal via afferent pathways to the central nervous system (CNS), processing and integration of the signal by the various centers of the CNS, and motor response resulting in muscle activation for the performance of various tasks and joint stabilization [5]. Proprioception is the process occurring along the afferent pathways of the sensorimotor system. It is defined as the acquisition of stimuli by peripheral mechanoreceptors (such as joint motion, position, velocity, length and tension of tissue) and the conversion of these mechanical stimuli into a neural signal that is transmitted along the afferent pathways to the CNS for processing [5].

A joint injury or joint disease, e.g., a knee injury or knee osteoarthritis (OA), can lead to a disturbance in the sensory system. This disturbance can be measured by proprioceptive acuity. Several studies have concluded that subjects with a knee injury or knee OA have impaired proprioception [6-13]. Two common measures of proprioception are kinesthesia, e.g., the threshold to detection of a passive motion (TDPM), and joint position sense (JPS), e.g., the active reproduction test. The TDPM is the most established test, is more reliable, and more sensitive in detecting differences between groups, such as between patients with anterior cruciate ligament (ACL) injury and uninjured controls, than measures of JPS [6,7]. A relation between impaired kinesthesia, measured by TDPM, and poor functional performance (measured by the one-leg hop test for distance, or balance in single-limb stance) and poor subjective outcome (measured by disease-specific questionnaires or subjective estimation of extremity function on a visual analog scale) has been found in patients with knee injury or knee OA [9,14-18]. Thus, kinesthesia may be an important indicator of the result of knee injury or knee disease.

When evaluating kinesthesia or the effects of intervention on kinesthesia, the assessment tools used need to be reliable. The two components of measurement error are systematic bias, e.g., learning or fatigue effects during the test, and random error due to inherent subject or instrument variation. To obtain sufficient information about the assessment tool, it has been recommended that several statistics be used; i.e., relative reliability, analysis of systematic change in the mean, and absolute reliability [19-22]. The intraclass correlation coefficient (ICC), which includes the systematic bias, can be used to assess relative reliability [20-22]. However, one disadvantage of the ICC is that it provides a value between 0 and 1, which is difficult to interpret clinically. To detect whether there is a systematic change in the mean, the paired t-test or mean difference between test and retest with a 95% confidence interval (CI) can be used [21,22]. Methods used to describe absolute reliability include calculations expressing the actual units of measurement, such as the Bland and Altman 95% limits of agreement (LOA) [21-23]. The LOA provide a 95% range of error for individuals, i.e., a real change in an individual's performance (e.g., before and after intervention) would be outside the LOA. The smaller the range, the more sensitive the method is in detecting change [19,23,24].

The aim of the present study was to assess the test-retest reliability of a device designed to measure knee proprioception, specifically the TDPM, in uninjured men and women.

## Methods

### Subjects

Twenty-four individuals (14 women and 10 men) with no history of neurological disease or major orthopedic lesions were included in the study. The sample size was based on the recommendations of Fleiss, i.e., that 15 to 20 subjects would be required for estimating the reliability of a quantitative variable [25]. The subjects' mean age was 41 years (SD 7.9 years), mean height 174 cm (SD 8.4 cm), mean weight 74 kg (SD 12.6 kg), and median activity level 4 (quartiles 4 to 5, range 2 to 9) according to the Tegner activity level scale, equal to moderately heavy work or recreational sports such as jogging, bicycling, or cross-country skiing [26]. The Research Ethics Committee at Lund University approved the study. All subjects gave their written informed consent to participate in the study.

### Kinesthesia test

**Figure 1:**
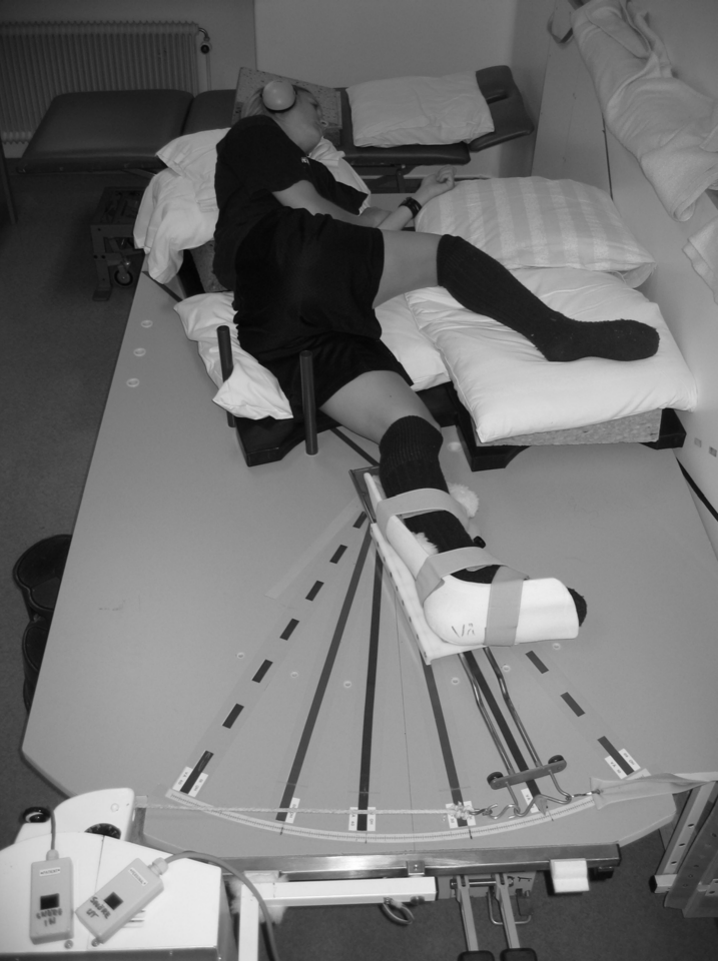
**Knee kinesthesia tested in a lateral decubitus position on a specially designed platform**. The subject is a model who did not participate in the study.

Kinesthesia was measured in a specifically designed apparatus, which has been described and used previously on patients with ACL injury and uninjured subjects [16,17,27-29]. The apparatus consists of a large rectangular platform. A new platform, mounted inside a steel frame has been constructed in order to make the device easier to use for older subjects (the platform was previously placed on the floor) (Figure 1). Mounted at one end is an electric motor with a wire. The wire is connected to a movable T-shaped sled to which a plastic splint is attached for fixation and positioning of the lower limb and foot. A metal bar is attached to the center of the sled, and pulling the wire in either direction causes the sled to rotate like the hand of a clock along the natural arc of extension or flexion of the knee. The arrow-shaped tip of the sled points to an analog scale on the platform (i.e., goniometer) to record movements in increments of 0.25°. The use of ball-bearings allows movements with little friction.

The subject lies in a lateral decubitus position, with the lower leg in the plastic splint. The splint supports the posterolateral part of the leg, but also has a slight anterior curve (to avoid valgus stress at the knee). The oversized construction allows for differences in the girth of the lower leg. Two bars mounted on the platform serve as guides for placing the thigh and trunk in a standard position, with the hip joint semiflexed. The knee joint was carefully positioned at the center of rotation. Markings on the platform allow accurate positioning of the knee in the different starting positions of knee joint flexion: 20° and 40°. Zero degrees is defined as full extension. The upper thigh and hip rest on a foam pillow (which can be adjusted to different heights, due to more extreme varus/valgus angulations), and pillows were also placed under the back to help the subject relax during the test. Care was taken to reduce any external stimuli of limb movement except those from the knee joint and surrounding structures. To minimize cutaneous sensations during the tests, all subjects wore short pants and a thick woolen sock, and the knee had no contact with the underlying surface. Visual cue of the leg was reduced by the subject's position, and closed eyes during the test, and auditory impulses were reduced during the threshold trial by earmuffs and a tape recorder playing a sound imitating the motor.

Measurements of the TDPM were performed towards extension (TE) and flexion (TF) from the two starting positions, 20° and 40° knee joint flexion, giving the variables TE20, TE40, TF20, and TF40. The subjects were asked to close their eyes, concentrate on their knee and respond (by raising their hand) when they felt any sensation of movement in their knee. The tape recorder was then turned on and, after a delay of 5 to 15 seconds (this information was not given to the subjects), the motor started to move the leg at a calibrated angular velocity of 0.5°·s^-1^. When the subject responded, the assessor stopped the motor and the movement was registered in degrees. The median values of three consecutive measurements of TE20, TE40, TF20, and TF40 were determined [16,17,27-29]. Higher values indicate poorer proprioceptive acuity [17]. The subjects were tested twice (test 1 and test 2), at about the same time of day with an interval of approximately one week, median value 7 days (quartiles 6–7, range 2–12 days).

The different starting positions were chosen so as to be within the working range of the knee during ordinary weight-bearing activities/exercise. Since the range of motion may differ between individuals (e.g., some individuals may have an extension deficiency), the most extreme joint positions were excluded. Thus, the tension in the muscles, capsule and ligaments was kept below high levels to avoid more variable tissue tensions between individuals, and to allow the subjects to relax without having their leg forced to maximum extension.

A slow speed was chosen to ensure that the subjects could not detect a sudden onset of motion and to maximally stimulate the joint receptors and minimize the contribution from muscle receptors. The tests were performed on both legs; the right leg being tested first, by shifting the apparatus arrangement from one side of the platform to the other.

### Statistical analysis

Since no differences were found between the men and the women, the results were analyzed together. No statistically significant difference was found between the variables in the right and left legs. To avoid the subjectivity in choosing one of the legs, the average of the right and left leg, i.e., (right+left)/2, for each variable was used for statistical analyses [30]. However, the results were confirmed using the results from the right and left legs separately in the analyses.

**Figure 2:**
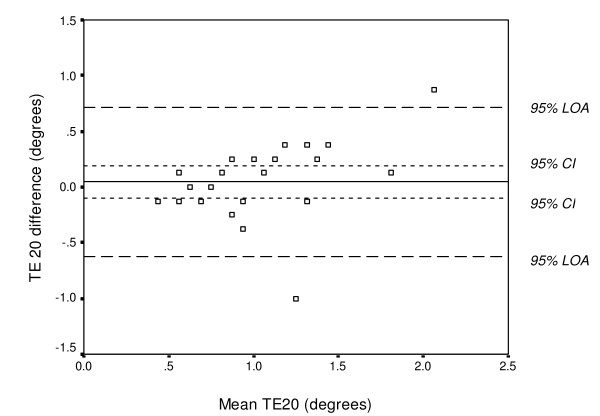
**Bland & Altman graph with limits of agreement (LOA)**. The differences between test sessions 1 and 2 (test 2 minus test 1) plotted against their mean for each subject for TE20 (degrees) in 24 uninjured subjects, together with the 95% confidence interval (CI) and the 95% LOA. In this figure, the differences are generally increasing with their means (heteroscedasticity). *Note*: several subjects have the same value.

**Figure 3:**
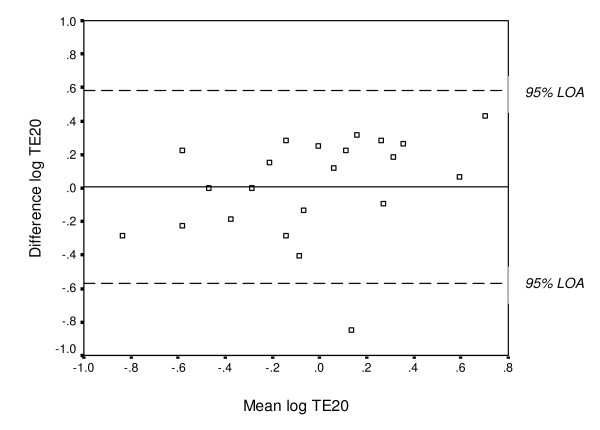
**Bland & Altman graph with limits of agreement (LOA) after log transformation**. The differences between test sessions 1 and 2 (test 2 minus test 1) plotted against their mean for each subject for TE20 after log_*e *_transformation with the 95% LOA. *Note*: several subjects have the same value.

A number of statistical methods of assessing test-retest reliability were used: 1) mean difference and 95% CI, 2) the two-way random effect model (absolute agreement definition), single measure ICC and 95% CI (ICC_2,1 _according to Shrout & Fleiss [31]), and 3) the Bland and Altman method of assessing agreement for individual subjects, which includes a scatter plot of the differences between test 1 and test 2 (test 2 minus test 1) against their mean with 95% limits of agreement (LOA) (i.e., mean difference ± 1.96 SD_diff_) [23]. Systematic bias can easily be estimated from these "Bland & Altman plots", e.g., if the values from the second test are greater than the values from the first test, the mean difference between the tests will be positive, and if the values from the second test are smaller than the values from the first test, the mean difference between the tests will be negative. If zero is included in the 95% CI, no significant systematic change in the mean is present. The plots also show indications of heteroscedasticity, i.e., larger variability for higher test values. In such cases, spreading out of data for larger values will be observed in the plots. Heteroscedasticity can be revealed by calculating a correlation coefficient between the absolute difference and the average of the test sessions. Performing a logarithmic transformation decreases this relationship. Without this log-transformation of heteroscedastic data, the LOA will be wider apart than necessary for low values and narrower than they should be for larger values [24]. Since heteroscedasticity was found in the present data (i.e., spreading out of data for larger values with a significant relationship between the absolute difference and the average of the test sessions), which is exemplified in Figure 2, a log-transformation (log_*e*_) was applied prior to calculation of the LOA (exemplified in Figure 3). The log-transformed LOA were then back-transformed (antilogged), giving values that can be interpreted in relation to the original scale. Using this transformation, the limits of the ratio of the two tests (LOA_ratio_) were obtained [24]. For example, a LOA_ratio _ranging between 0.80 and 1.20 times means that one test may differ from another by 20% below, i.e., a 20% decrease in TDPM, to 20% above, i.e., a 20% increase in TDPM in an individual.

## Results

### Test-retest reliability

The mean (SD) for test sessions 1 and 2, mean difference (SD_diff_) with 95% CI, ICC values, and 95% LOA_ratio _between test 1 and test 2, are given in Table 1. The mean difference and 95% CI revealed no statistically significant difference between test 1 and test 2 in three of the four kinesthetic variables (zero is included in each interval). For one kinesthetic variable (TF20), the values from test 2 were lower than the values from test 1. TDPM would need to decrease between 10% (TE20) and 38% (TF20), and increase between 17% (TF40) and 24% (TE40) in groups of uninjured subjects to be 95% confident of detecting a real change. ICC values between 0.59 and 0.70 were found in the kinesthetic variables, except for TE40 (ICC 0.16). The TDPM variables obtained with a starting position of 20 degrees (TE20 and TF20) tended to have higher ICC values and narrower LOA_ratios _than those from the 40-degree starting position (TE40 and TF40).

**Table 1:** Test-retest reliability in the kinesthetic variables, in 24 healthy subjects.

**Kinesthetic variables**	**Test session 1 Mean (SD)**	**Test session 2 Mean (SD)**	**Mean difference (SDdiff),** ** 95% CI**	**ICC_**2,1**_** **(95% CI)**	**95% LOA_**ratio**_**
TE20	0.98 (0.37)	1.03 (0.51)	0.05 (0.35), -0.10 – 0.19	0.70 (0.42 – 0.86)	0.57 – 1.78
TE40	1.20 (0.52)	1.26 (0.75)	-0.06 (0.84), -0.41 – 0.30	0.16 (-0.27 – 0.53)	0.31 – 3.09
TF20	1.55 (0.66)	1.29 (0.70)	-0.26 (0.55), -0.49 – -0.03	0.63 (0.31 – 0.83)	0.42 – 1.57
TF40	0.79 (0.28)	0.81 (0.34)	0.02 (0.28), -0.10 – 0.14	0.59 (0.25 – 0.80)	0.51 – 2.01

Mean (SD) for test sessions 1 and 2, mean difference (SD_diff_), 95% confidence interval (test 2 minus test 1), intraclass correlation coefficient (ICC_2,1_) (95% confidence interval), and 95% limits of agreement (LOA) of back-transformation (antilog) of the LOA on a log scale (LOA_ratio_) in the kinesthetic variables towards extension (TE) and flexion (TF) from the starting positions 20° and 40° (TE20, TE40, TF20, TF40).

## Discussion

The reliability of the proprioceptive device has been assessed in a previous study by Fridén et al. [29]. However, in that study, only the systematic change in the mean was used to assess test-retest reliability [29]. If several reliability statistics are used, this may provide us with information regarding whether some variables are more reliable than others, and if the assessment tool is reliable for groups of subjects and for individual subjects. We found that three kinesthetic variables (TE20, TF20 and TF40) were sufficiently reliable to observe change in groups of subjects (ICC values ranging from 0.59 and 0.70, and 95% CI ranging from 10% to 38%), but that relatively large differences in an individual's performance would be required to confidently state that a real change had taken place. The TDPM variables from the 20-degree starting position seemed to be more reliable than those from the 40-degree position.

No systematic change in the mean was noted in three of the four kinesthetic variables (TE20, TE40, TF40), as zero was included in the 95% CI. This is in line with the previous study by Fridén et al. [29]. The values of TF20 from test 2 were significantly lower than the values from test 1 (zero is not included in the 95% CI), which may be interpreted as a learning process. However, since the 95% CI was quite close to zero (Table 1), the clinical relevance of this learning effect can be questioned. According to the recommendations of Fleiss [25], ICC values above 0.75 represent excellent reliability, values between 0.4 and 0.75 represent fair to good reliability, while values below 0.4 represent poor reliability. Three variables (TE20, TF20, and TF40) showed ICC values above 0.40 but below 0.75, indicating good reliability, while one variable (TE40) showed poor reliability (ICC 0.16). Large variations between subjects result in high ICC values and, thus, more homogeneous data would result in lower ICC values [22]. However, the standard deviations of the mean values of TE40 were not markedly smaller than those of the other three variables (Table 1). Thus, the low ICC value for TE40 cannot be explained by more homogeneous data. The high ICC values for TE20, TF20, and TF40, indicate that these variables are likely to observe change in groups of subjects without pathology. These high ICC values for TDPM variables are supported by findings in other studies [6,32]. To be 95% confident of detecting a real change in groups of subjects, TDPM would need to decrease (i.e., improve) between 10% (TE20) and 38% (TF20), and increase (i.e., decline) between 17% (TF40) and 24% (TE40). In previous studies, patients with ACL injury had over 30% higher TDPM values (i.e., poorer kinesthetic acuity) than uninjured subjects [27,28].

To evaluate changes over time in an individual, the magnitude of the change must exceed the inherent variability of the measurements. The LOA can be used to assess a "real" change in an individual's performance as a result of, for example, intervention, i.e., if the difference between two measurements is outside the LOA, there is a true change in performance [21]. Since heteroscedasticity was found in the data, a log-transformation and a back-transformation were performed, giving the limits of the ratio between the two tests (LOA_ratio_) [24]. In the kinesthetic variables, TE20 showed the narrowest LOA_ratio_, ranging between 0.57 and 1.78 times, i.e., one test may differ from another by 43% below (i.e., 43% lower value) to 78% above (i.e., 78% higher value). The TE40 showed the widest LOA_ratio_, ranging between 0.31 and 3.09 times, i.e., one test may differ from another by 69% below to 209% above. The LOA_ratios _were all rather wide, indicating that these tests cannot detect small changes in an individual's performance, i.e., a substantial difference in an individual's measurements would be required to confidently state that a change had actually taken place. According to Rankin and Stokes [22], at least 50 subjects are needed in reliability studies, otherwise the 95% limits of agreement will be too wide. Thus, one reason for the wide LOA_ratios _in our study may be a too small sample size. To evaluate TDPM over time in individuals, it may be important to calculate LOA in a larger group of subjects (n ≥ 50). We have found no studies reporting absolute reliability of TDPM variables for knee kinesthesia. However, in a study by Pincivero et al. [33], the intra-subject variation was assessed for knee proprioception, by measuring the ability of subjects to "catch their leg" when the knee was dropped into extension from a relaxed position. They also found relatively large intra-subject variation, using SEMs as measures of absolute reliability [33]. Thus, proprioceptive tests may be more useful and appropriate when distinguishing between groups of subjects, such as patients and controls, or when investigating the effect of an intervention in a group of subjects.

The different starting positions when measuring the TDPM were chosen to be within the working range of the knee during ordinary weight-bearing activities/exercise. The tendency towards higher ICC values and narrower LOA_ratios _for the TDPM variables with the starting position at 20 degrees than those from 40 degrees, suggests that the variables TE20 and TF20 may be more reliable. Several other studies have reported higher reliability and/or higher sensitivity in detecting movements, in proprioceptive variables close to the end range of motion compared with in the mid range of motion in patients with ACL injury and uninjured subjects [18,29,33]. These findings may be explained by an increased afferent impulse generation near the terminal joint position, which is required to protect the joint from injury [4]. Thus, from the results of the present study and those of others [18,29,33], it can be argued that measurements of TDPM close to the end range of motion are probably the most reliable and sensitive.

## Conclusion

Three kinesthetic variables (TE20, TF20 and TF40) were found to be reliable in observing change in groups of subjects. TDPM would need to decrease between 10% and 38%, and increase between 17% and 24% in groups of uninjured subjects to be 95% confident of detecting a real change. The LOA_ratios _revealed that small changes in an individual's measurements cannot be detected, i.e., a relatively large difference in an individual's kinesthetic measurements would be required to confidently state that a real change had taken place. These tests may thus be more useful and appropriate for observing change in groups of subjects. The higher ICCs and narrower LOA_ratios _in the TDPM variables obtained with the starting position at 20 degrees knee joint flexion (i.e., closer to terminal extension), indicate that these variables are more reliable than those obtained with the starting position at 40 degrees. We, therefore, recommend that the TDPM variables from 20 degrees be used in future studies on subjects without pathology.

## Competing interests

The author(s) declare that they have no competing interests.

## Authors' contributions

EA contributed to the design of the study, analysis and interpretation of the data, and drafting and critical revision of the manuscript. JF and JL collected the data and assisted in writing the manuscript. All authors read and approved the final version.

## Pre-publication history

The pre-publication history for this paper can be accessed here:


